# Messenger RNA and Plasmid DNA Vaccines for the Treatment of Cancer

**DOI:** 10.3390/vaccines13090976

**Published:** 2025-09-14

**Authors:** Jena E. Moseman, Daeun Shim, Donghwan Jeon, Ichwaku Rastogi, Kaitlyn M. Schneider, Douglas G. McNeel

**Affiliations:** Department of Medicine, Carbone Cancer Center, University of Wisconsin-Madison, 1111 Highland Avenue, Madison, WI 53705, USA; jemoseman@wisc.edu (J.E.M.); dshim6@wisc.edu (D.S.); donghwan.jeon@wisc.edu (D.J.); irastogi@wisc.edu (I.R.); kmschneider6@wisc.edu (K.M.S.)

**Keywords:** cancer immunotherapy, cancer vaccines, RNA vaccine, DNA vaccine

## Abstract

Immunotherapy is now an established therapy for nearly a third of patients with cancer. Most therapies, typically using cytokines or checkpoint blockade therapy, rely on global activation of immune effector cells. The ability of vaccines to activate specific populations of cells has led to a renewed interest in their ability to treat cancers, either alone or with other immune therapies or other conventional therapies. The COVID-19 pandemic sparked a new interest in nucleic acid vaccines with the development of new technologies and the short manufacturing time for vaccine implementation. Nucleic acid-based cancer vaccines have been studied for decades, but have shown modest anti-tumor efficacy as monotherapies, as many of these vaccines encode for shared tumor-associated antigens (TAAs) and must overcome immune tolerance. New developments, technologies, routes of delivery, and combination therapies have paved the way for new approaches and clinical trials involving nucleic acid vaccines for the treatment of cancer. Here we review mRNA and pDNA vaccines for the treatment of cancer, including similarities and differences in their mechanisms of action, an overview of these treatment modalities in preclinical and clinical studies, methods to improve these vaccine strategies, and exciting new combination approaches in development.

## 1. Introduction to Nucleic Acid Vaccines for the Treatment of Cancer

Cancer incidence and mortality remain a major global concern, with 20 million new cases diagnosed and 9.7 million deaths in the year 2022 alone [1]. For many cancers, the first line of treatment is surgery, followed by radiotherapy and/or chemotherapy. However, it is estimated that approximately 90% of cancers develop resistance [2,3]. Resistance to primary treatment underscores the need for new therapies or combination therapies. One treatment modality that has been gaining popularity in recent years is the field of immunotherapy, and cancer vaccines in particular.

Cancer vaccines are a way to “train” the body’s immune system to recognize and kill the tumor. Cancer vaccines developed as a concept in the late 1800s when Dr. William Coley observed tumor regressions after patients were injected with inactivated bacteria [4,5]. Since then, numerous types of cancer vaccines have been developed and are reviewed elsewhere, including viral vector-based [6], bacterial-based [7], protein or peptide-based [8], cell-based (including transfection of antigen presenting cells with mRNA for cell therapy) [9], and nucleic acid vaccines using messenger RNA (mRNA) or plasmid DNA (pDNA, hereafter referred to simply as “DNA”). Since viral and bacterial-based vectors rely on attenuated pathogens to enter cells and encode proteins, a simpler and safer way to deliver the genetic material to cells is needed. Nucleic acid vectors can serve as an efficient and safe alternative for the delivery of genetic material, which will be the focus of this review article.

Nucleic acid vaccines are advantageous to various cancer vaccine platforms due to their cost, ease of manufacture, and safety profile. The first DNA vaccine to enter human clinical trials was a vaccine targeting HIV in 1998 [10]. Although DNA vaccines were deemed safe from these initial trials, it was unclear whether these vaccines mounted significant clinical efficacy. Similarly, mRNA vaccines have a long history. Unlike DNA, mRNA cannot efficiently be directly delivered to cells, due to the inherent instability of the molecule and the presence of RNases in blood and tissues [11]. Hence, the first “mRNA vaccines” were based on the concept of transfecting and transferring dendritic cells with mRNA into the body for protein translation, with the first human clinical trial for cancer therapy based on this technology conducted in 2003 (NCT00003432) [12]. These initial studies, as well as preclinical studies in mice, eventually paved the way for direct injection of synthetic mRNA using modified nucleotides and lipid nanoparticles to evade immune detection, prevent degradation, and improve efficacy [13]. However, the true advancement of both mRNA and DNA vaccines came with the COVID-19 pandemic, where the first mRNA and DNA vaccines were approved in humans for the prophylactic infectious disease setting [14,15]. Although nucleic acids vaccines have proven highly successful in the infectious disease setting, there are still many challenges and issues that remain to be addressed for these vaccines to have clinical efficacy in the cancer setting.

## 2. Mechanisms of Action of DNA Versus mRNA Vaccines

### 2.1. Mechanisms of Cellular Uptake and Antigen Presentation

DNA and mRNA vaccines both elicit immune responses by enabling host cells to produce antigenic proteins that can activate the immune system [16]. However, the cellular mechanisms underlying their delivery, uptake, processing, and antigen presentation differ due to the distinct nature of DNA and RNA molecules. Both DNA and mRNA vaccines rely on delivering genetic material into host cells, often using lipid-based nanoparticles or other vectors to facilitate cellular entry [17].

mRNA vaccines are typically directly introduced into the cytoplasm of target cells using either lipid nanoparticle carriers or electroporation. Following cellular uptake, which commonly occurs via endocytosis of mRNA-LNP complexes, the mRNA is released into the cytoplasm, where it is translated into the target antigen by ribosomes [18]. Ionizable lipids play a crucial role in this process, as they enable the delivery vehicle to escape the endosomal compartment by undergoing a conformational change at the lower pH of the endosome. This “dumping” of mRNA into the cytoplasm ensures efficient protein translation [19]. Once the protein is synthesized in the cytosol, it can be broken down into peptides via the endogenous pathway and presented on MHC class I molecules to activate CD8+ T cells [20]. mRNA can also be taken up by professional antigen-presenting cells (APCs) such as dendritic cells (DCs), which process and present the antigen via both MHC class I and MHC class II pathways, allowing for the activation of both CD8+ and CD4+ cytotoxic and helper T cells [21]. Classical antigen presentation by professional APCs occurs when exogenous antigen is processed and presented on MHC class II molecules, activating CD4+ helper-T cells. However, cross-presentation, a mechanism where exogenously produced antigens can be presented on MHC class I molecules, can occur if the mRNA is taken up and expressed by non-professional APC, and the protein is take up and presented by dendritic cells to activate CD8+ T cells [22].

In contrast, DNA vaccines require an additional step of entering the cell nucleus for transcription [23]. After uptake, typically via endocytosis, DNA must successfully traverse the cytoplasm and cross the nuclear membrane to access the transcriptional machinery [24]. This necessity for nuclear delivery is a key distinction from mRNA vaccines [25]. Once inside the nucleus, the DNA is transcribed into mRNA, which then follows the same pathway as mRNA vaccines—translation in the cytoplasm, antigen production, and subsequent presentation on MHC molecules. However, the need for nuclear entry can pose a barrier, reducing the overall efficiency of antigen production compared to mRNA vaccines [16]. Additionally, the instability of mRNA in vivo may reduce its effectiveness as an immune modulator, whereas DNA is known to be stable for over six months in vivo [26]. mRNA’s instability also causes it to be degraded rapidly, resulting in short bursts of protein expression, potentially causing a need for frequent booster immunizations. One trafficking study using eGFP mRNA reported peak mRNA expression 8 h after immunization, and peak protein expression 24 h after immunization [27].

While both mRNA and DNA vaccines can be taken up by a variety of cell types, including myocytes and fibroblasts at the site of injection, professional APCs, such as dendritic cells, macrophages, and B cells, are the most critical for the initiation of adaptive immune responses [28]. mRNA vaccines are particularly adept at targeting DCs, especially when delivered in nanoparticle formulations designed to enhance uptake by these cells [29]. The rapid translation of mRNA in the cytoplasm makes it a highly efficient platform for inducing robust immune responses, particularly when aimed at activating both cellular and humoral arms of the immune system [30]. In contrast, DNA vaccines may be taken up by a broader range of cell types, but due to their requirement for nuclear entry, the efficiency of antigen presentation may vary, especially in non-dividing cells where nuclear access is more limited [31]. B cells have been shown to be the primary professional APC that can uptake and process the naked DNA plasmid to present the encoding antigen, however B cells require licensing by DCs to enable antigen presentation [32,33]. To enhance uptake by APCs and improve immunogenicity, DNA vaccines often rely on additional strategies, such as electroporation, to facilitate more effective nuclear delivery [34].

A key aspect of both mRNA and DNA vaccine delivery systems is the use of ionizable lipids, which assist in endosomal escape [35]. Ionizable lipids are neutral at physiological pH but become positively charged in the acidic environment of the endosome, destabilizing the endosomal membrane and facilitating the release of their cargo into the cytoplasm [36]. This mechanism is particularly important for mRNA vaccines, where protection from nucleases and release into the cytoplasm are necessary for efficient antigen expression [37]. The same strategy can be applied to DNA vaccines, though the DNA must again be transported to the nucleus after cytoplasmic release, adding an extra step to the antigen production process [38,39].

In summary, the key distinctions between DNA and mRNA vaccines lie in the requirement for DNA to reach the nucleus versus the cytoplasmic localization of mRNA, and in the mechanisms of delivery, processing, and antigen presentation. Both platforms have their unique advantages, but the more direct cytoplasmic pathway of mRNA may offer a more rapid and efficient means of inducing immune responses.

### 2.2. Mechanisms of Innate Immune Recognition

Cells have inherent processes to recognize mRNA and DNA in the cell and send out “danger signals” when these molecules are recognized, generating an innate immune signaling cascade. The key sensors that recognize foreign RNA in the endosomes are Toll-like receptors (TLR) 3 and TLR 7/8 [40]. There are also cytosolic RNA sensors, such as RIG-1 and MDA5, that play a role in RNA detection and result in an innate immune signaling cascade [40]. Since detection of foreign mRNA and activation of these sensors may result in the destruction of the mRNA molecule, more recent efforts have shifted to allow for modified mRNA that can evade immune detection by these sensors. This has enabled the modern mRNA-LNP vaccines to successfully deliver their cargo without undergoing immune destruction [41,42,43]. In contrast, there is a greater quantity of DNA sensors inside the cell compared to mRNA. In the endosomes, the major DNA sensor is TLR9 [44]. There are also many cytosolic DNA sensors, such as cGAS/STING, AIM2, IFI16, the DExD/H-box helicase family members, RNA polymerase 3, DAI, DNA-PK, and MRE-11 [44]. In general, cellular recognition of foreign DNA leads to a pro-inflammatory response, characterized by the secretion of pro-inflammatory cytokines and type I interferon [44]. While conceivable that these differences in intracellular RNA and DNA sensors could affect the nature of the immune response generated by these different vaccine approaches, this has not been rigorously assessed.

### 2.3. Types of Immune Responses Generated by Immunization with mRNA or DNA Vaccines

While no direct comparisons of the resulting immune responses generated by mRNA and DNA vaccines have been made, there have been reports of the types of immune responses generated by each of these types of vaccines. In the case of mRNA cancer vaccines, there have been reports of strong cell-mediated immunity, characterized by the generation of CD4+ and CD8+ antigen-specific immune responses [45,46,47,48]. Similarly, cancer DNA vaccines have been reported to generate cellular immune responses across multiple studies [49]. Notably, many studies using DNA vaccines to encode tumor antigens have also demonstrated humoral immune responses [49]. Although more studies and trials have been conducted using DNA vaccines, both DNA and mRNA vaccines appear to elicit cellular and humoral immunity. The magnitude, phenotype, and longevity of these cellular responses are unknown, though. Insights regarding direct comparisons of mRNA and DNA will be beneficial in determining whether these treatment modalities are functioning similarly or whether there may be an advantage to combining DNA vaccines and mRNA vaccines for the treatment of cancer.

## 3. mRNA and DNA Vaccines in Preclinical Animal Models Encoding the Same Antigens

Targets for tumor vaccines can be categorized into tumor-associated antigens (TAAs) and tumor-specific antigens (TSAs). TAAs are “shared” self-antigens which may be aberrantly expressed or overexpressed in tumor cells. In general, due to mechanisms of central and peripheral tolerance, TAAs are generally characterized by low immunogenicity, and T cell receptors often have low affinity for these antigens [50]. Therefore, nucleic acid vaccines encoding TAAs may require additional methods to enhance their immunogenicity. These strategies include incorporating immunoadjuvants, optimizing administration delivery methods and routes, employing prime-boost regimens, or encoding multiple antigens to target tumor heterogeneity [51]. In contrast, most TSAs result from aberrant antigen expression (e.g., frame-shift mutations or individual point mutations) within the tumor cells resulting in mutation-associated neoantigens (MANA) [52]. Since these antigens would not have undergone thymic tolerance, they tend to elicit slightly stronger immune responses and may be more readily targeted through vaccination, at least in murine models [53].

While many preclinical studies have utilized mRNA and DNA vaccines for the treatment of cancer, no studies have directly compared mRNA and DNA vaccines in the same study. Therefore, here we will focus on studies that have examined either mRNA or DNA encoding the same antigen as a means of comparing their actions. Table 1 highlights those studies that have used either mRNA or DNA targeting the same antigen in similar tumor models. Only preclinical studies involving the direct administration of nucleic acid vaccines into the subjects—excluding those utilizing ex vivo DC loading—were considered. Specific target antigens are described in detail below.

### 3.1. Mucin 1 (MUC1)

MUC1 is a transmembrane glycoprotein on epithelial cells, with aberrant glycosylation linked to malignant cellular transformation. Aberrant expression of MUC1 has been found in several types of cancer, including gastric, breast, ovarian, and bladder cancers. Therefore, MUC1 has been one of the major targets for cancer vaccine development [75]. Various nucleic acid vaccines encoding MUC1 have been developed to elicit anti-tumor response against MUC1-expressing tumors in murine models. An early study of prophylactic MUC1 plasmid DNA (pMUC1) displayed limited efficacy in inducing tumor protection in a murine colorectal cancer model. Co-administration of a plasmid encoding the murine IL-18 was needed to enhance the tumor protection, memory response, and epitope spreading [54]. However, a therapeutic MUC1 DNA vaccine, pcDNA3.1-MUC1 (20 μg, delivered by gene gun and electroporation), elicited enhanced anti-tumor responses in the murine colorectal model. Immunization promoted IFNγ and granzyme B secretion by CD8+ T cells and enhanced their proliferation and cytotoxicity against the target murine colorectal cells in vitro and in vivo [55].

Efforts to improve the efficacy of DNA vaccines have included strategies such as developing plasmid DNA that encodes multiple tumor antigens to address tumor heterogeneity. A DNA vaccine encoding MUC1 and survivin fusion antigens, VR-MS, increased in vitro killing of MUC1+ survivin+ B16 cells and significantly delayed tumor growth using a 100 μg dose [57]. Additional strategies to improve DNA vaccines have been investigated, such as enhancing the immunogenicity of plasmid DNA by designing fusion plasmids that encode multiple TAAs and immunoadjuvants. CpDV-IL2-MS is a bicistronic DNA plasmid encoding survivin, MUC1, and IL-2 [58]. In a study by Liu et al., BABL/c mice with MUC1+ survivin+ colorectal and lung tumors were immunized with CpDV-IL2-MS, CpMS+IL-2, or PBS to examine immunological and antitumor effects of the fusion plasmid. Mice immunized with 100 μg CpDV-IL2-MS had equivalent IFN-γ secretion, tumor cell cytotoxicity, tumor growth rates and survival compared to mice treated with CpMS+IL-2 [58]. Furthermore, incorporation of soluble PD-1 into the plasmid (CpDV-IL2-sPD1/MS) elicited a greater number of cures in mice bearing colorectal or lung cancers [76]. Similarly, mice receiving 100 μg of a chimeric MUC1 DNA vaccine, encoding the transmembrane- and C-terminal domain-deleted Muc1 gene fused to the human HSP70 gene, had greater anti-tumor effects. Immunization with the MUC1 vaccine in the murine B16/MUC1+ melanoma model induced greater MUC1-specific CD8+ T cell response compared to HSP70 or deletion of the C-terminal domain alone [56].

Although still in its early stages, research using mRNA vaccines encoding MUC1 is also underway. Mice inoculated with 4T1 mammary carcinoma cells had enhanced IFN-γ secretion and MUC1-specific CD8+ T cell killing when immunized with 10 μg of a MUC1 mRNA nanovaccine encapsulated in lipid/calcium/phosphate nanoparticles (LCP) compared to mRNA or LCP alone. However, additional combination of MUC1 LCP-mRNA with an anti-CTLA4 antibody was needed to enhance CD8+ T cell recruitment, inhibit the tumor-promoting STAT3 pathway, and further enhance anti-tumor responses in the murine triple negative breast cancer cells, in vivo [59].

### 3.2. Melanoma Antigen Family A (MAGE-A)

MAGE-A is a cancer-testis antigen (CTA) found in multiple cancer types such as melanoma, brain, breast, lung and ovarian cancers [77]. Unlike most TAAs, CTAs are considered highly desirable due to their distinct characteristics, including restricted expression in immune-privileged organs, tumor-specific expression, and widespread presence across various types of cancers [78]. Immunization by electroporation with 30 μg of a DNA plasmid encoding MAGE-A3 isoform significantly enhanced Th1 (IgG2a, IFN-γ) and Th2 (IgG1, IL-4) responses, and delayed tumor growth when compared to mice immunized with empty vector in the murine lung cancer LLC model. These responses were further augmented when the plasmid was modified to encode MAGE-A3 and soluble PD-1 [60]. While many studies have targeted MAGE-A3, tumor cells can express two or more MAGE-A antigens [79]. Duperret et al. analyzed the Cancer Genome Atlas and found that patients across tumor types commonly expressed multiple MAGE-A isoforms [61]. Therefore, this group designed a synthetic consensus MAGE-A DNA vaccine encoding murine MAGE-A1, -A2, -A3, -A5, -A6, and -A8. Splenocytes from immunized mice displayed IFN-γ response and exhibited high cytolytic potential (CD107a+ T-bet+ IFN-γ+) when stimulated with MAGE-A peptides in vitro. In an inducible melanoma model, driven by Cre activation in melanocytes via tamoxifen induction, immunization using 25 μg DNA, delivered by electroporation, resulted in delayed tumor growth, reduced tumor invasion depth in the skin, and increased infiltration of CD8+ T cells with an activated phenotype (CD44+ PD-1+) [61].

In a study by Choi et al., investigators immunized groups of mice with MAGE-A3 mRNA encapsulated in various lipid nanoparticles, and inoculated mice with CT26 colorectal cancer cells two weeks later. Prophylactic use of 20 μg of MAGE-A3 mRNA encapsulated in various lipid nanoparticles significantly delayed tumor growth, limited lung and liver metastases, and prolonged survival compared to untreated control mice. Researchers repeated this study but administered lipid nanoparticle encapsulated MAGE-A3 mRNA post-tumor inoculation. Therapeutic vaccination post implantation of CT26 cells also delayed tumor growth, comparable to a standard chemotherapeutic agent doxorubicin, but without weight loss that occurred with chemotherapy. The vaccine enhanced humoral responses (IgG2a and IgG2b), although no significant changes in IFN-γ or IL-4 levels were observed in the sera. In vitro co-culture of splenocytes with tumor lysates did increase IL-4, although IFN-γ remained unchanged [62].

### 3.3. Human Papillomavirus (HPV)

HPV is one of the most prevalent sexually transmitted infections and accounts for approximately 5% of all cancer cases worldwide. The virus has been associated with cervical, vulvar, vaginal, anal, and head and neck cancers [80]. An FDA-approved prophylactic HPV vaccine is estimated to hold the potential to prevent more than 90% of cancers caused by HPV [81]. However, for patients with existing HPV-positive cancers, therapeutic approaches are being developed to target HPV antigens, particularly the oncogenic viral early protein E6 and E7, using DNA and mRNA vaccines. Mice bearing HPV-associated TC-1 tumors were immunized by gene gun with 16 μg of plasmid DNA encoding HPV16 E7 fused to calreticulin (CRT) to enhance MHC-I presentation. Immunization induced a robust E7-specific CD8+ T cell response, resulting in CD8+ T cell-dependent protection against the tumor and pulmonary nodules. Moreover, the inclusion of CRT triggered antiangiogenic activity, which contributed to the reduction in pulmonary tumor nodules [63]. Similarly, other studies have included a related immunoadjuvant, heat shock protein (HSP) 70, to enhance immunogenicity of the E7 encoding plasmid DNA. While the administration of E7 vaccine alone delayed tumor growth compared to the PBS or empty vector controls, the tumor growth suppression was enhanced when co-administered with a vector encoding HSP70. Immunization increased lymphocyte proliferation and in vitro cell-mediated cytotoxicity [64]. These findings underscore the importance of an effective adjuvant, such as CRT or HSP70, to augment the immune responses of HPV-targeting immunotherapies. To illustrate this, further enhancement of the plasmid DNA design, by encoding a fusion protein product consisting of signal peptide fused to *Mycobacterium tuberculosis* HSP70 and HPV16 E7 (with a point mutation to eliminate oncogenic potential), increased the number of IFN-γ secreting E7-specific CD8+ T cells in the spleen and induced complete in vivo protection against TC-1 tumors [65]. The plasmid design was also further improved by Peng et al. to optimize codons for expression and encode 4 total HPV antigens: HPV16 E6, HPV16 E7, HPV18 E6, and HPV18 E7. Mice immunized with 25 μg of this vaccine (pBI-11) had an increased number of HPV-specific IFN-γ-secreting CD8+ T cells. Immunization of TC-1 tumor-bearing mice also had reduced tumor growth [66].

HPV vaccines have also been developed using mRNA approaches. Immunization with 10 μg of mRNA-LNP encoding HPV E7 expanded HPV-specific CD8+ T cells in the spleen and blood of mice implanted with syngeneic HPV+ oropharyngeal squamous cell carcinoma (mEERL) cells [67]. Subsequent single-cell RNA sequencing (scRNA-seq) analysis comparing vaccinated mice to those immunized with PBS or an empty vector revealed distinct clustering patterns in T cells of the spleen and tumors of vaccinated mice. Vaccination enhanced innate immune activation in the spleen and promoted both effector differentiation and exhaustion of CD8+ T cells in the tumor microenvironment. Coupled with scTCR-seq, the vaccine induced clonal expansion primarily in tumor-infiltrating effector and exhausted cell subclusters. Therefore, the HPV mRNA-LNP vaccine drove effector cells toward an exhaustion trajectory, marked by elevated expression of both effector and inhibitory markers, which play crucial roles in mediating antitumor immunity [67]. Other HPV-targeting mRNA vaccines also demonstrated similar anti-tumor efficacy. HPV16 E6/E7-based mRNA-LNP immunization led to significant tumor regression within the first week of treatment in mice bearing the HPV16+ cervical cancer model. Suppression of tumor volume and complete responses were found to be dose-dependent [68]. Similarly, bi-weekly immunization of mice with 30 μg of mHTV-02, encoding HPV-16 and HPV-18 E6/E7 mRNA-LNP, induced an antigen-specific immune response, indicated by increased secretion of IFN-γ and IL-2, and frequency of IFN-γ+ and TNF-α+ CD8+ T cells [69]. Immune cell infiltration and anti-tumor effects were examined in mice with HPV16 E6/E7-positive TC-1 tumors that were vaccinated with mHTV-02 weekly. mHTV-02 immunization increased the infiltration of multifunctional (IFN-γ+ GzmB+) CD8+ T cells and effector memory CD4+ and CD8+ T cells to the tumor. Additionally, vaccine-induced regression of tumor growth and prolonged survival was observed [69].

### 3.4. Kirsten Rat Sarcoma Viral Oncogene Homolog (KRAS)

KRAS is a small GTPase that transduces signals from receptor tyrosine kinases to downstream pathways, regulating cell proliferation, differentiation, and transformation. Therefore, KRAS represents one of the commonly mutated proto-oncogenes, especially at the G12 residue, found in lung, pancreatic, and colon cancers [82]. While the KRAS G12C mutation has been a successful target, through small molecule inhibitors such as sotorasib and adagrasib, there has been less success targeting other G12 mutations [83]. However, there have been recent attempts to target the KRAS G12 variant through nucleic acid vaccines. Immunization using a plasmid DNA encoding the inactive mutant KrasG12DN17, a dominant-negative mutant devoid of oncogenic activity, produced significant therapeutic responses against a murine lung adenocarcinoma model (TetO-Kras4b^G12D^/Scgb1a1-rtTA). Mice were immunized with 100 μg Kras^G12D^ DNA vaccine intramuscularly at five-day intervals, administered a total of ten times. Anti-tumor response was indicated by a decreased number of lung nodules and enhanced expression of IFN-γ, IL-12 and IL-4 in splenocyte s [70]. As an attempt to achieve broad-spectrum anti-tumor immunity, researchers designed an mRNA encoding all prevalent RAS variants (mRNA-1521). Groups of mice were immunized with mRNA-1521 or PBS on days 0, 21, and 49 and then inoculated with CT26 colon cancer cells on day 56. Groups receiving vaccine prophylactically had increased infiltration of CD8+ T cells and granzyme B+ CD8+ T cells, and delayed growth of colorectal cancer CT26 in vivo [72]. A therapeutic study of mRNA similarly encoding multiple KRAS variants increased tumor-infiltration of CD8+ T cells and significantly delayed tumor growth in the syngeneic KRAS G12C-expressing lung cancer LL/2-bearing mice [73].

### 3.5. Summary of Preclinical Models Encoding the Same Antigens

In summary, while no studies to our knowledge have directly compared mRNA and DNA cancer vaccines, both types of vaccines have been able to successfully elicit cellular and humoral responses in murine preclinical and in vitro models, with increased cytokine secretion, and decreased tumor growth reported in most studies. Of note, higher doses of DNA, administered using alternative delivery methods such as gene gun devices or electroporation, were required to generate sufficient anti-tumor responses. This is not surprising given the requirement for DNA to enter the nucleus to be transcribed prior to translation. In contrast, in general, mRNA could be delivered in lower doses without assisted delivery devices, likely due to the protection of mRNA encapsulated in lipid nanoparticles along with the ability for direct translation in the cytoplasm.

## 4. Monotherapies of mRNA and DNA Vaccines in Human Clinical Trials

Given the breadth and success of preclinical studies utilizing mRNA or DNA vaccines, many of these therapies have been translated to human clinical trials as cancer treatments. While DNA vaccines have largely been explored in clinical trials of breast, cervical, prostate, and melanoma cancers, mRNA vaccines have been predominantly tested in pancreatic and non-small cell lung cancers. Notably, target antigens of DNA vaccines were mostly TAAs, whereas many mRNA vaccines were designed to target patient-specific MANA or neoantigens such as KRAS G12D, G12V, G12C, or G13D mutations. Because of these distinctions, and the fact that very few clinical trials have been conducted using mRNA or DNA encoding the same antigens, we have focused on the clinical trials that have used mRNA or DNA vaccines as monotherapies, with or without adjuvants. Details of these recent clinical trials are summarized in Table 2.

### Summary of Human Clinical Trials Using Monotherapy mRNA or DNA Vaccines

As highlighted in Table 2, many trials have been conducted with single-agent mRNA or DNA vaccines, delivered by different routes or methods, at many different doses, and with or without the use of adjuvants. However, within specific cancer types, none of these trials have used an mRNA or DNA vaccine encoding precisely the same antigen(s). Therefore, only general conclusions can be drawn from these studies in a particular disease.

In one trial, in which patients with melanoma received an mRNA vaccine encoding four shared antigens, NY-ESO-1, MAGE-A3, tyrosinase, and TPTE, 3 out of 25 patients exhibited partial responses and 7 of 25 patients had stable disease [94]. Additionally, a patient receiving monotherapy who had a partial clinical response also had a detectable antigen-specific immune responses [94]. While there is not a DNA vaccine encoding these exact four TAAs, three studies have reported on DNA vaccines encoding tyrosinase. These studies have demonstrated safety and tolerability (NCT00698100), antigen-specific immune responses in 6/15 patients [91], and antigen-specific immune responses in 11/26 patients [92].

In general, whether the vector was mRNA or DNA based, many of these clinical trials exploring monotherapy vaccination noted antigen-specific CD4+ or CD8+ T cell responses. However, while these were mostly early phase trials without robust clinical endpoints, only a few of these clinical trials demonstrated any evidence clinical benefit, regardless of whether the vaccine vector was mRNA or DNA. These underwhelming clinical trial results underscore the need for better optimization of the route of delivery, stability of the molecule, targeting to antigen presenting cells, or the need of combination therapies to overcome mechanisms of immune resistance.

## 5. Methods to Improve mRNA and DNA Vaccines as Monotherapies

Although nucleic acid vaccines are a promising approach for the treatment of cancer, their efficacy as monotherapies, as demonstrated from Table 2, is largely still lacking, potentially due to failure to mount a sufficient immune response, failure to reach antigen presenting cells, or the development of mechanisms of treatment resistance. Therefore, efforts to improve immunogenicity of nucleic acid vaccines by construct design, enhanced delivery methods, or combination therapies are currently underway and described below.

### 5.1. Methods to Improve mRNA Vaccine Efficacy

Of recent interest in the mRNA field is self-amplifying mRNA. Self-amplifying mRNA encodes viral replication machinery in addition to the gene of interest, resulting in mRNA that will replicate itself in vivo following uptake [110]. These vectors are considered safer than traditional viral vector-mediated vaccines since the virus itself is not introduced and will not replicate [110]. As previously stated, the instability of mRNA causes it to be rapidly degraded, leaving the need for frequent booster immunizations. Self-amplifying mRNA vaccines have the potential to be delivered less frequently than standard mRNA viruses and in lower doses [110]. Preclinical cancer studies using self-amplifying mRNA and LNPs are somewhat limited; however, one study by Maine et al. developed a self-replicating mRNA vaccine platform (SMAART). CT26 tumor-bearing mice were immunized with 10 μg of LNP SMARRT RNAs on days 3 and 17 after tumor injection [111]. SMARRT vaccination induced polyfunctional CD4+ and CD8+ T cells and reduced tumor growth in mice with colorectal cancer. Additionally, rhesus masques with influenza were immunized with SMARRT encoding HA from different strains of influenza on days 0 and 56 at varying doses (1, 10, and 100 ug). The vaccine generated polyfunctional CD4+ and CD8+ T cells in non-human primates at all doses [111]. Another study examined a single immunization with gDE7 mRNA-LNP in an HPV-16-associated tumor model [112]. Mice were inoculated with TC-1 cells and immunized with 30 μg of gDE7 vaccine 3 days later. The study found that a single immunization with a self-replicating mRNA vaccine could control tumor growth in mice [112]. Although research in this area is still ongoing, one self-amplifying mRNA vaccine was approved for protection against COVID-19 in Japan [113].

Another area that is gaining interest in the mRNA field is the manipulation of lipid nanoparticles to increase targeted delivery to certain tissues. One major problem with standard LNP formulations is that many of these LNPs traffic to the liver, or cells that take up RNA/LNP formulations traffic to the liver. While this may be beneficial for diseases of the liver or cancers with liver metastases, many other malignancies may not benefit from liver trafficking. Therefore, recent efforts have focused on LNP modifications to target specific tissues of interest. The selective organ-targeting lipid nanoparticle (SORT) technique involves adding a fifth molecule to the standard four-component LNP to target specific organs such as lung, spleen, and liver [114]. This technology has been used in preclinical cancer vaccines shown in a study by Chen et al. where researchers found a certain LNP, 113-012B, improved uptake in the draining lymph nodes instead of the liver in comparison to ALC-0315 [115]. Mice were subcutaneously injected at the tail base with 113-012B and luciferase mRNA (mLuc) to track in vivo distribution of expressed protein. The intensity of mLuc six hours after injection was higher in draining lymph nodes of mice that received 113-012B compared to other LNPs.

Finally, several groups have modified LNPs to increase their intrinsic ability to act as adjuvants or by directly incorporating adjuvants into the LNP or mRNA-LNP constructs. These methods have been extensively reviewed elsewhere in the past [116,117,118]. However, new studies have demonstrated that modifying the structures or ratios of the non-ionizable lipid components can affect vaccine immunogenicity and intrinsic adjuvanticity [119,120]. While these studies have largely been conducted in infectious disease models, these methods can conceivably be adapted to mRNA vaccines encoding tumor antigens for the treatment of cancer.

### 5.2. Methods to Improve DNA Vaccine Efficacy

Plasmid DNA vaccines, in general, have low immunogenicity when they are delivered passively by needle-syringe through intradermal, intramuscular, or subcutaneous routes. Therefore, several groups have focused their efforts on improving delivery methods to enhance DNA entry into the cells rather than rely on passive uptake. One of these methods is electroporation, which incorporates an electric pulse immediately after the needle-syringe delivery, allowing for the creation of pores in cell membranes to deliver DNA [121,122]. Several groups have reported increased transfection using electroporation. Broderick et al. found increased humoral immunity in addition to increased protein expression when utilizing a needleless piezoelectric device for electroporation in guinea pigs [123]. Electroporation has also been evaluated for DNA vaccines in human clinical trials; however, a study by Chudley et al. demonstrated similar immunological responses in patients immunized with or without electroporation [124]. Another study by Low et al. showed increased antibody responses in patients receiving a DNA vaccine plus electroporation in comparison to DNA vaccine alone in a clinical trial of prostate cancer [125]. Although some of these studies showed potential positive therapeutic responses, vaccination with electroporation has been shown to cause increased pain at the injection site [122]. This has led to multiple groups focusing their efforts on improving DNA vaccine delivery methods in other ways such as by gene gun or needless devices.

Needleless immunization methods are increasing in popularity due to better tolerability by patients. Gene gun delivery of plasmid DNA was initially implemented in the 1990s to deliver DNA to plant cells, which are notoriously difficult to transfect [126,127]. This technology involves a device, the gene gun, which delivers metal microparticles coated with nucleic acid with high velocity to penetrate the cell membrane, usually propelled by a gas such as helium [128]. Since the initial studies in plants, gene gun delivery has successfully been implemented in mammalian cell and animal studies [129,130,131,132,133,134]. These promising preclinical studies led to human clinical trials, and DNA vaccines delivered by gene gun have proven to be safe, well-tolerated, and immunogenic, with doses of DNA as low as 1 µg being demonstrated to elicit cellular and humoral immunity [135,136]. Similar safety and immunological efficacy results have been shown in human clinical trials using needless fluid-based “jet” devices [137,138]. The only DNA vaccine approved for use in humans is delivered using a needle-free jet device by PharmaJet, indicating the safety, tolerability, and efficacy profiles of needle-free delivery devices are promising for future plasmid DNA delivery trials [139].

## 6. Combination Therapies Using mRNA or DNA Vaccines

Due to the safety and immunogenicity observed in most small early-phase clinical trials, but lack of profound clinical efficacy, several groups have explored strategies combining nucleic acid vaccines with commonly used therapies or immune-modulating therapies in preclinical studies or human clinical trials. A summary of ongoing human clinical trials is presented in Table 3.

### 6.1. Combination Therapies with Radiotherapy

In addition to its direct cytotoxic effects, radiation can indirectly induce immunogenic changes in the tumor microenvironment. As radiation-induced DNA damage accumulates in tumor cells, mutations and genomic instability increase, leading to immunogenic cell death. The release of various DAMPs (e.g., HSP, ATP, CRT, and high mobility group box 1) activates macrophages and dendritic cells to enhance antigen presentation. Additionally, the expression of vascular adhesion molecules increases, and inflammatory cytokines and chemokines such as IL-1β, IL-23, and CXCL-10 are locally released. In surviving cancer cells, radiation can activate the cGAS/STING pathway to release type I IFN, further promoting the release of pro-inflammatory molecules, activation of dendritic cells and lymphocytes, and increased susceptibility to CD8+ T cell-mediated cytotoxicity. This cycle ultimately enhances the priming and activation of cytotoxic CD8+ T cells, leading to increased tumor cell killing [154,155,156,157]. However, radiation can also induce immunosuppressive effects, including the recruitment of Tregs and MDSCs as well as the promotion of M2-like macrophages, which may contribute to resistance to both radiation and immunotherapy [158,159]. Therefore, combining radiation with nucleic acid vaccines may enhance anti-tumor efficacy, but may also require careful consideration of factors such as the radiation type, radiation treatment regimen, and the timing of therapies.

In one combination study, an E7-encoding RNA lipoplex (RNA-LPX) was administered to mice inoculated with HPV-related cancers, followed by 12Gy of local external beam radiation [160]. E7 RNA-LPX immunization increased the frequency of intratumoral E7-specific CD8+ T cells, while radiation reduced tumor mass and hypoxia, enhancing the tumor killing capacity of antigen-specific CD8+ T cells. This combination approach delayed tumor growth and prolonged survival of mice bearing TC-1 and C3 tumors. The response depended on the total radiation dose, as both 12Gy and an equivalent biological effective dose of 3 fractions of 6Gy similarly enhanced anti-tumor immunity when combined with E7 RNA-LPX [160].

Another study using a two-component mRNA cancer vaccine, consisting of free and complexed OVA-encoding mRNA, was tested in combination with external beam radiotherapy in mice bearing E.G7-OVA tumors [161]. Two vaccinations were administered per week, starting on the first day of radiation, with a total radiation dose of 6Gy delivered in three equal fractions over three consecutive days. The combination delayed tumor growth and prolonged survival compared to either treatment alone. A total of 5/7 complete responders were noted when rechallenged with parental OVA negative EL4 cells, suggesting the establishment of broad immune protection potentially through antigenic spread. Gene expression analysis revealed that the combination treatment significantly downregulated genes associated with tumor-associated factors and upregulated tumor suppressor-related genes compared to immunization alone [161].

Using a similar ovalbumin-based model, we have explored radiopharmaceutical therapy (RPT) targeting the lipid rafts of tumor cells, ^90^Y-NM600, as a strategy to improve the efficacy of anti-tumor DNA vaccines. The adoptive transfer of naïve OVA_257-264_-specific CD8+ T cells before or after low dose ^90^Y-NM600 administration did not enhance anti-tumor response in mice bearing E.G7-OVA-PD-L1^hi^ tumors. However, when the tumor-specific CD8+ T cells were activated in vivo using OVA-encoding plasmid DNA in combination with ^90^Y-NM600, tumor growth was significantly delayed. Notably, DNA vaccine was most effective when delivered prior to ^90^Y-NM600. Our findings suggest that tumor-specific CD8+ T cells need to ideally be present and activated prior to RPT for greater anti-tumor efficacy, suggesting that anti-tumor vaccination should be delivered prior to RPT [162]. While these studies using artificial OVA models cannot be directly compared due to differences in radiation modalities, they serve as proof of concept that the combination of radiation and nucleic acid vaccines enhances anti-tumor efficacy compared to either treatment alone.

Other studies have explored the combination of radiation with HPV-targeting nucleic acid vaccines. In one study, a single dose of 14Gy of external beam radiation was delivered to mice bearing E7-expressing TC-1 tumors, followed by three doses of CRT/E7(detox), a DNA construct encoding HPV16 E7 (modified to abrogate function but retain conformation) and fused to calreticulin (CRT) to enhance antigen presentation. The combination significantly delayed tumor growth and prolonged survival compared to either treatment alone. Additional timing studies revealed that when radiation was given at the time of the second dose of vaccination, significantly higher numbers of E7-specific IFN-γ+ CD8+ T cells were present in the splenocytes and the tumor [163].

Very few clinical trials are currently exploring the use of radiation therapy in combination with nucleic acid vaccines. However, a clinical trial by Papachristofilou et al. examined combination therapy of an mRNA vaccine (300 μg of CV9202, encoding six non-small cell lung cancer antigens) along with 20Gy local radiation over the course of 57 days. Patients that received CV9202 and local radiation had immune responses to the six encoded antigens and increased antigen-specific CD4+ and CD8+ T cells over time [153]. Collectively, these studies using nucleic acid vaccines with radiation therapy suggest that future preclinical and clinical studies should focus on how radiotherapy parameters (e.g., radiation type, dose, schedule) affect combination strategies with vaccination, as well as assess the mechanisms of potential synergy.

### 6.2. Combination Therapies with Immune Checkpoint Inhibitors (ICI)

Blocking antibodies against the immune checkpoints PD-1, PD-L1, and/or CTLA-4 have been approved by FDA for the treatment of squamous cell head and neck cancer, melanoma, Merkel cell carcinoma, cutaneous squamous cell carcinoma, hepatocellular carcinoma, renal cell carcinoma, cervical cancer, small cell lung cancer, non-small cell lung cancer, triple negative breast cancer, gastric carcinoma, gastroesophageal junction carcinoma, Hodgkin lymphoma, advanced or metastatic urothelial cancer, primary mediastinal B-cell lymphoma, and cancers bearing microsatellite instability or mismatch repair deficiency [164]. However, despite approval for use in these multiple types of cancer, only a small percentage of these patients respond to immune checkpoint inhibitors as monotherapies. Moreover, response to therapy is generally restricted to those cancers with evidence of pre-existing activated tumor-reactive T cells. This suggests they may be best utilized in combination with other therapies, such as cancer vaccines, that can selectively activate tumor-specific T cells.

Multiple groups are studying mRNA vaccines in combination with immune checkpoint inhibitors. Jin et al. created a targeted mRNA vaccine incorporating a cGAS agonist into the LNP formulation to augment immunogenicity. In a LLC-OVA murine model, mice treated with the mRNA vaccine and PD-L1 blockade had reduced tumor growth, likely due to increased infiltration of cell immune cell types into the tumor and increased pro-inflammatory cytokines [165]. Similarly, Wang et al. encapsulated 9 μg of Trp2 mRNA and 1 μg of PD-L1 siRNA into lipid calcium phosphate nanoparticles. Mice inoculated with B16F10 melanoma tumors were treated once, when tumors were ~300 mm^3^ in size, with this vaccine or mock encoding controls. Mice receiving antigen-specific mRNA in addition to PD-L1 siRNA had reduced tumor growth [166]. Another group has shown similar trends using a PD-1 blocking antibody in combination with an ovalbumin-encoding mRNA-nanostructured lipid nanocarrier (LNC) vaccine in B16-OVA and E.G7-OVA tumor models. Mice were inoculated with tumors (B16-OVA) on day 0 and were immunized with OVA LNC-mRNA (3 μg) on days 3, 6, and 9. Additionally, mice received anti-PD-1 on days 7, 12, and 17. Combination therapy-treated mice exhibited prolonged survival and a greater number of cures than monotherapy-treated control mice [167]. These successful preclinical models have paved the way for several clinical trials combining mRNA vaccines with immune checkpoint inhibitors, and these have shown signs of efficacy in patients with pancreatic ductal adenocarcinoma [148] and melanoma [140]. In particular, a clinical trial targeting individualized melanoma neoantigens (mRNA-4157, V940) given with PD-1 blockade (pembrolizumab), in comparison to PD-1 blockade alone, showed prolonged recurrence-free survival and improved distant metastasis-free survival in patients with resected high-risk melanoma [140]. These positive study results led to “breakthrough therapy” designation by the FDA for this therapy. Other early-phase clinical trials investigating mRNA vaccines in combination with checkpoint inhibitors are underway (Table 3).

Several preclinical studies evaluating DNA vaccines in combination with ICIs have also been conducted. A study by Lopes et al. showed that combination therapy of a DNA vaccine encoding the P815A tumor antigen plus antibodies blocking PD-1 and CTLA-4 prolonged survival and reduced tumor growth in murine P815 mastocytoma [168]. The combination of vaccine and ICI also promoted IFNγ, IL2, and granzyme B production in the tumor microenvironment, suggesting enhanced activation of antigen-specific T cells [168]. Similar preclinical studies in mice have shown improvement in DNA vaccine efficacy when delivered in combination with checkpoint blocking antibodies against PD-1, CTLA-4, LAG-3, or a combination thereof, in mouse models of melanoma [169,170], multiple myeloma [171], prostate cancer [172], glioblastoma [173], human papillomavirus-positive cancers [61], and colorectal cancers [174,175]. As most of these preclinical studies have been conducted using DNA vaccines, it remains unknown whether there are differences between mRNA and DNA vaccines when combined with ICI blockade or whether one approach is more effective than the other when combined with ICI blockade.

As depicted in Table 3, several clinical trials are investigating the use of DNA vaccines in combination with checkpoint inhibitors targeting PD-1, PD-L1, CTLA-4, and/or LAG-3. Most of these trials remain underway. However, two separate trials have been reported using a DNA vaccine encoding a prostate tumor antigen, prostatic acid phosphatase (PAP, pTVG-HP) in patients with early recurrent prostate cancer or more advanced castration-resistant, metastatic prostate cancer (mCRPC) [144,176]. In the trial of patients with mCRPC, vaccination was given either concurrently with PD-1 blockade (pembrolizumab), or PD-1 blockade was administered after completing a 12-week course of vaccination. Objective responses and PSA declines were observed when treatment was given concurrently but not sequentially, suggesting that vaccination is best delivered with concurrent ICI blockade [143]. Concurrent administration of this DNA vaccine and PD-1 blockade (nivolumab) in patients with early recurrent prostate cancer similarly resulted in PSA declines and prolonged stable disease [144].

### 6.3. Combination Therapies with Toll-like Receptor Agonists

mRNA and DNA vaccines have an inherent ability to act as self-adjuvants due to the ability to activate pattern-recognition receptors (PRRs), including toll-like receptors (TLRs) and RIG-I-like receptors. However, many studies suggest that there may be additional benefits to adding these innate immune sensor ligands as vaccine adjuvants [177]. While lipid-based nanoparticles have been primarily utilized to prevent mRNA degradation and improve delivery to antigen-presenting cells, some have also been shown to activate TLR4 signaling and thereby increase anti-tumor vaccine efficacy in murine tumor models [178,179]. In one of these studies, an optimized C1 nanoparticle was used to encapsulate ovalbumin-encoding mRNA. In vitro studies using bone marrow derived dendritic cells (BMDCs) revealed increased activation of BMDCs as demonstrated by increased protein expression of CD40, CD80, CD86, as well as increased secretion of IL-1β, IL-6, and IL-12p70 in comparison to controls, following treatment with this nanoparticle. However, BMDCs from Tlr4^−/−^ mice failed to show these effects when treated with C1-mRNA, indicating the BMDC activation was dependent on TLR4 signaling [179]. In a study conducted by Lee et al., a TLR1/2 agonist was incorporated into lipid nanoparticles encapsulating an ovalbumin-targeting mRNA vaccine. This resulted in better suppression of E.G7-OVA tumor growth in mice [180]. Similarly, an ovalbumin-specific mRNA vaccine administered with a TLR 7/8 agonist also remarkably improved the expansion of ovalbumin-specific CD8+ T cells and their infiltration into ovalbumin-expressing murine lymphoma and prostate cancer, resulting in significant suppression of tumor growth [181]. Haabeth et al. demonstrated that combination of TLR9 agonist and an OVA-encoding mRNA vaccine increased cytotoxic T cell responses and led to complete regression of A20-OVA lymphoma in mice [182].

Similarly, co-administration of DNA vaccines with a TLR3 or TLR7 agonist showed a greater expansion of HPV E7-specific CD8+ T cells than DNA treatment alone, and the combination also induced significant regression of TC-1 tumors [183]. Vaccination of mice with a DNA vaccine encoding the ligand-binding domain of the androgen receptor, a prostate tumor associated antigen, and combined with TLR3 and TLR9 agonists, resulted in secretion of type 1 IFN from antigen-presenting cells and suppression of TRAMP-C1 prostate tumor growth [184].

Human clinical trials are also beginning to investigate the use of TLR agonists in combination with nucleic acid vaccines. Notably, in cervical cancer, one study is combining a TLR7 agonist (Imiquimod) with a DNA vaccine encoding E6 and E7 (NCT00788164), based on encouraging preclinical data in murine tumor models [185].

### 6.4. Combination Therapies with Chemotherapy

Chemotherapy is a standard treatment for many cancers, but it is often associated with immunosuppressive effects [186]. However, certain chemotherapeutic agents have been shown to modulate the immune system in ways that can enhance the efficacy of cancer vaccines, creating an opportunity for combinatorial approaches to overcome the limitations of each treatment [187]. Some chemotherapeutic agents, such as cyclophosphamide and doxorubicin, have immunomodulatory effects that make them suitable for combination with mRNA and DNA vaccines [188,189]. These drugs can deplete immunosuppressive cell populations, increase tumor antigen release and/or enhance antigen presentation. For example, low-dose cyclophosphamide, a DNA alkylating agent, has been shown to selectively reduce populations of regulatory T cells (Tregs) and regulate myeloid-derived suppressor cells (MDSCs) infiltration, both populations known to inhibit the immune response in the tumor microenvironment, in a dose-dependent manner [190,191,192]. Cytotoxic chemotherapies also lead to the destruction of tumor cells, which can cause the release of tumor-associated antigens (TAAs) and neoantigens [193]. Tumor cell death induced by chemotherapy also results in the release of damage-associated molecular patterns (DAMPs), which further stimulate immune responses [194]. Certain chemotherapies can upregulate the expression of MHC class I molecules on tumor cells, making them more susceptible to recognition by cytotoxic T lymphocytes (CTLs) [195]. Additionally, chemotherapy can promote dendritic cell maturation, enhancing their ability to present vaccine-derived antigens to T cells [196].

Preclinical studies combining mRNA or DNA vaccines with chemotherapy have demonstrated enhanced antitumor effects compared to either treatment alone [197,198]. In a murine study using E.G7-OVA tumor model, mice were treated with an mRNA vaccine (encoding ovalbumin, 32 μg) alone or in combination with cisplatin (1.2 mg/kg) or docetaxel (25 mg/kg). The results from this study demonstrated improved tumor control in the combination group when using either of the chemotherapeutic agents [161]. The combination approach resulted in significant tumor regression and extended survival compared to monotherapy treatments alone. In another pre-clinical study using a genital tract TC-1 tumor model, Bialkowski et al. demonstrated that the combination of E7-TriMix mRNA vaccine (2.5 μg of each TriMix component and 5 μg of E7 antigen, administered on day 8 and 13) with cisplatin (4 mg/kg, administered on days 8, 9, 15, and 16) caused an increase in E7-specific T cells within tumors, and secretion of IFNγ, compared to the spleen. Additionally, the E7-targeting mRNA vaccine and chemotherapy led to complete regression of cervical tumors in murine models [199].

In preclinical studies of DNA vaccines, combining DNA vaccination with cyclophosphamide has shown promise. In one study, mice were inoculated with 4T1 breast tumor cells on day 0, followed by 50 μg immunizations of a plasmid DNA vaccine encoding fibroblast activating protein-α (CpVR-FAP) into both the right and left tibia muscles on days 2, 9 and 16 post tumor implantation [200]. Cyclophosphamide was administered (50 mg/kg) on days 1, 8 and 15 post tumor implantation. This study demonstrated that chemotherapy enhanced the recruitment of CD8+ T cells and improved the overall efficacy of DNA vaccines in tumor-bearing mice, as evidenced by decreased tumor growth and prolonged survival in the combination therapy group in comparison to either monotherapy alone. Additionally, the proportions of Tregs in the spleen, and immunosuppressive cytokines such as IL-10 and CXCL-12 in the tumor microenvironment, were decreased [200]. Notably, DNA vaccines in combination with cyclophosphamide led to a more durable immune response, without impairing antigen specific immunity resulting from the vaccination. Similarly, Chen et al. also demonstrated synergistic effects of paclitaxel, a chemotherapeutic drug that acts as a mitotic inhibitor, with a chimeric DNA vaccine encoding E7 antigen and connective tissue growth factor, when delivered in low doses frequently over a long period of time [198]. Mice were inoculated with TC-1 tumor cells, and then immunized weekly with 2 μg of pcDNA3-CTGF/E7, delivered by gene gun. Paclitaxel was administered either daily or two times per week at doses of 3, 6, and 25 mg/kg. Mice that received the combination treatment of DNA vaccine plus either 3 mg/kg or 6 mg/kg paclitaxel resulted in smaller tumor volumes and prolonged survival. Additionally, mice that received DNA vaccine in combination with 6 mg/kg paclitaxel had greater numbers of antigen-specific T cells detected in the spleen in comparison to the other combination treatment doses tested [198]. Another group tested the timing of chemotherapy with respect to DNA vaccine. Mice were inoculated with TC-1 cells on day 0, followed by treatment on day 8 with either PBS, cisplatin alone (10 mg/kg, days 8 and 11), DNA vaccine alone (2 μg of CRT/E7 delivered by gene gun with boosters on days 12 and 16), DNA vaccine followed by cisplatin 4d later (DNA on d8, 12, and 16, with cisplatin on days 20 and 23), or cisplastin followed by DNA vaccine 4d later (Cisplatin on d8 and 11, DNA on d15, 19, and 23). The mice that received cisplatin prior to DNA vaccine, in comparison to the other groups tested, had reduced tumor growth and increased numbers of antigen-specific CD8+ cells in their spleens and tumors [163].

In human clinical trials, studies are underway in breast cancer that combine DNA vaccines with chemotherapy. Although these studies are not yet completed, and preclinical data are limited, these initial studies suggest at least the feasibility of combining mRNA or DNA vaccines with chemotherapy. However, several challenges must be considered for these combination therapies. As demonstrated in the studies above, different timing regimens of the DNA or chemotherapy agents, or the dosing strategy, may affect tumor growth, survival, and antigen-specific T-cell generation. Therefore, future studies will need to refine the timing, dosing, and selection of chemotherapeutic agents to maximize synergy.

## 7. Concluding Remarks and Future Directions

mRNA and DNA vaccines each have advantages and disadvantages. mRNA is rapidly degraded, has transient protein expression, and generally requires cold storage, which may limit its use in certain countries. DNA has the requirement of needing to reach the nucleus of antigen-presenting cells, which may mean higher doses, electroporators, or alternative delivery methods may need to be used to achieve the immunogenicity required to control tumor growth. Unfortunately, there have been no head-to-head studies in preclinical models or human trials using mRNA or DNA vaccines that encode the same antigen, and thus it remains difficult to determine whether one approach is preferred. Due to differences in uptake, cellular processing, recognition by different intracellular nucleic acid sensors, and antigen expression kinetics, it can be speculated that vaccination with mRNA or DNA may produce some differences in the phenotype and magnitude of immune responses generated. This will be an area of future research, to understand the optimal means to improve the anti-tumor efficacy of each approach.

Notwithstanding, vast improvements in technology, delivery methods, and combination therapies have enabled nucleic acid vaccines to be more immunogenic than when they were first implemented for cancer treatments. It is expected that these newer approaches with greater immunogenicity will lead to greater anti-tumor efficacy in clinical trials. Newer gene delivery methods (including needless delivery methods and the use of self-amplifying constructs), the encoding of multiple antigens, the use of different adjuvants, and the use of nucleic acid vaccines in combination with other agents, are mostly still underway and in early stages of development, but have already shown promising results in a small number of clinical trials. In particular, clinical trials incorporating combination treatments of nucleic acid vaccines with checkpoint blockade and/or chemotherapy have already proven successful across many cancer types as evidenced by detectable antigen-specific T cell responses, prolonged recurrence-free survival, prolonged distant metastasis-free survival, and increased tumor infiltration of T cells (Table 3). Many of these studies have been conducted using PD-1, PD-L1, or CTLA-4 blocking antibodies; however, the field of immune checkpoint blockade is rapidly evolving and new therapies targeting other checkpoints, such as LAG-3, VISTA, TIM-3, or TIGIT, or a combination of these, may have more success when used in combination with nucleic acid vaccines. Furthermore, future directions using strategies to overcome the presence and function of regulatory immune cells in the tumor microenvironment may be necessary to further improve the anti-tumor efficacy of nucleic acid vaccines.

## Figures and Tables

**Table 1:** mRNA and DNA vaccines in preclinical animal models encoding the same antigens.

Antigen	Vaccine (Vector)	Nucleic Acid	Route and Dose	Response	Tumor Model	Reference
MUC1	pMUC1 (Vical 1055)	DNA	ID: 3 doses, biweekly (10 μg)	• Did not induce tumor protection alone • Co-administration of plasmid encoding murine IL-18 was needed to induce tumor protection, memory response, and epitope spreading	Colorectal (MC38/MUC1+)	[54]
	pcDNA3.1-MUC1 (pcDNA3.1(-))	DNA	IM: 2 doses, biweekly (20 μg)	• Increased IFN-γ and granzyme B production • in vitro and in vivo cytotoxicity	Colorectal (CT26/MUC1+)	[55]
	MUC1-HSP70 (pcDNA3)	DNA	ID: 3 doses, weekly (100 μg)	• Increased CD8+ proliferation • Increased IFN-γ secretion • Increased in vitro and in vivo cytotoxicity	Melanoma (B16/MUC1+)	[56]
	VR-MS	DNA	IM: 3 doses, biweekly (100 μg)	• Increased in vitro cytotoxicity • Delayed tumor growth	Melanoma (B16/MUC1+survivin+)	[57]
	CpDV-IL2-sPD1/MS (pBluescriptIISK (+))	DNA	IM: 2 doses, biweekly (100 μg)	• Increased In vitro cytotoxicity • Increased antibody production • Increased IFN-γ secretion • Promoted tumor-infiltration of CD8+ T cells while reducing Treg infiltration • Delayed tumor growth and prolonged survival	Colorectal (CT26/MUC1+ survivin+) Lung (Lewis/MUC1+ survivin+)	[58]
	MUC1 mRNA (encapsulated in lipid/calcium/phosphate nanoparticle)	mRNA	SC: 2 doses, weekly (10 μg)	• Increased IFN-γ secretion • Increased in vivo MUC1-specific CD8+ T cell-dependent killing of tumor cells	Triple negative breast (4T1)	[59]
MAGE-A	pVAX1-MAGE-A3 (pVAX1)	DNA	IM: 3 doses, every week (30 μg) with electroporation	• Delayed tumor growth • Increased IFN-γ response by splenocytes • Enhanced Th1 (IgG2A, IFN-γ) and Th2 (IgG1 and IL-4) responses • Augmented anti-tumor responses when plasmid fused encode to soluble PD-1	Lung carcinoma (LLC)	[60]
	MAGE-A (encoding A1, A2, A3, A5, A6, A8)	DNA	IM: 3 doses, biweekly for in vitro; 4 doses, weekly for in vivo studies (25 μg) followed by electroporation	• Increased IFN-γ-secreting splenocytes • Increased frequency of IFN-γ+, CD107a+ T-bet+ IFN-γ+ and TNFα+ CD8+ T cells • Delayed tumor growth • Decreased tumor invasion depth in the skin • Increased tumor-infiltration of CD44+ PD-1+ CD8+ T cells	Inducible model of melanoma (Tyr::CreER;Braf^Ca/+^;Pten^lox/lox^)	[61]
	MAGE-A3 mRNA-containing LNP (DMKD/PS)	mRNA	IM: 2 doses, biweekly (20 μg)	• Delayed tumor growth and prolonged survival • Enhanced humoral response (IgG2a/b) • Did not change IFN-γ or IL-4 in sera • In vitro co-culture of splenocytes from with tumor increased IL-4 but not IFN-γ	Colorectal (CT26)	[62]
HPV	HPV16 E7/CRT (pDNA3)	DNA	Gene gun: 4 doses, weekly (16 μg)	• CD8+ T cell-dependent tumor protection • Reduced pulmonary tumor nodules and tumor growth • Decreased microvessel density in lung tumor nodules	HPV-associated lung (TC-1)	[63]
	pcDNA/E7, pcDNA/HSP70	DNA	IM: 2 doses, biweekly	• pcDNA/E7 delayed tumor growth compared to PBS and empty vector • Co-administration of pcDNA/HSP70 further delayed tumor growth, enhanced lymphocyte proliferation, in vitro cytolytic activity of splenocytes of immunized mice	HPV-associated lung (TC-1)	[64]
	pNGVL4a/Sig/E7(detox)/HSP70	DNA	IM (syringe needle and needle-free jet injection device): 2 doses, weekly (50 μg) Gene gun: 2 doses, weekly (2 μg)	• Increased the number of IFN-γ-secreting E7-specific CD8+ T cells in the spleen (gene gun generated the highest number while requiring the least dose) • Complete in vivo protection	HPV-associated lung (TC-1)	[65]
	pBI-11 (codon-optimized pNGVL4a/Sig/E7(detox)/HSP70)	DNA	IM: 3 doses, 3-day interval (25 μg)	• Increased number of HPV-specific IFN-y-secreting CD8+ T cells (splenocytes and PBMC) • Delayed tumor growth	HPV-associated lung (TC-1)	[66]
	HPV16 E7 mRNA-LNP	mRNA	SC, IV: 3 doses, 5-day interval (10 μg)	• Expansion of HPV-specific CD8+ T cells in the spleen and tumor • Spleen CD8 +T cells clustered in the IFN-induced group • Tumor-infiltrating CD8+ T cells clustered in effector memory and exhausted groups • Clonal expansion in tumor-infiltrated effectors and exhausted cell subclusters	HPV-associated oropharyngeal squamous cell carcinoma (mEERL)	[67]
	HPV16 E6/E7 mRNA-LNP	mRNA	IM or SC: 3 doses, weekly (10 μg or 20 μg); 1 or 2 doses, 10 day interval (3 μg or 10 μg)	• Tumor regression after the first week of treatment • Formation of immunological memory (rejection tumor upon re-challenge) • Responses were not dose-dependent and did not differ by injection route	HPV-associated cervical (C3.43)	[68]
	mHTV-02	mRNA	IM, IT, SC, IV, ID: 3 doses, weekly (6.25 μg, 12.5 μg, 25 μg)	• Increased secretion of IFN-γ and IL-2 • Increased HPV-specific IFN-γ+ and TNF-α+ CD8+ T cells • Increased tumor-infiltration of IFN-γ+ Granzyme B+ CD8+ T cells • Increased memory CD8+ and CD4+ • Tumor regression and prolonged survival • IM or IT significantly delayed tumor growth and prolonged survival, while IV did not affect growth or survival • Dose-responses observed for naïve immunized mice, but therapeutic doses as little as 6.25 µg were effective at controlling tumor growth	HPV-associated lung (TC-1)	[69]
KRAS G12	KrasG12DN17	DNA	Gene gun: 10 doses, 5-day interval (10 μg) and/or IM: 10 doses, 5-day interval (100 μg)	• Decreased number of lung nodule • Increased expression of IFN-γ, IL-12, IL-4 in the splenocytes of vaccinated mice • Combination of GG and IM immunization methods significantly increased CD8+ T cell infiltration into the lungs	Bi-transgenic inducible, spontaneous lung adenocarcinoma (CCSP-rtTA/Tet-Op-K-Ras4G12D)	[70]
	KRAS G12V mRNA	mRNA	3 doses, 5-day interval (10 μg)	• Increased the percentage of KRAS G12V-specific CD8+ T cells in the spleen • Increased IFNγ secretion by splenocytes • Delayed the growth of G12V-bearing tumors	Melanoma (G12V and HLA-A11:01 overexpressing B16F10)	[71]
	mRNA-1521	mRNA	Immunized on day 0, 21, and 49	• Tested for prophylactic use only • Delayed tumor growth • Combination with anti-PD-1 further delayed tumor growth • IgG response • Greater infiltration of CD8+ and Granzyme B+ CD8+	Colon carcinoma (CT26)	[72]
	mRNA encoding transcripts for multiple KRAS mutant ag	mRNA	Not specified	• Increased tumor infiltration of CD8+ T cells (slight 0.5-fold increase in CD44+ CD8+) • Decreased Treg infiltration • Attenuated tumor growth by 37%	Lung (LL/2)	[73]
Neoantigens	B_SARS_T_KPC-mRNA_Vax	mRNA	20 µg mRNA; immunized every 4 days for three doses, then every 3 days for two doses; total of 5 doses	• Significantly delayed tumor growth • Increased germinal center B cells • Increased T_FH_ cells, antigen-specific CD4 T cells and antigen-specific CD8+ T cells • Increased Granzyme B-producing CD4+ and CD8+ T cells	Pancreatic (KPC 6422)	[74]

**Table 2:** Monotherapy mRNA and DNA vaccines in human clinical trials.

Cancer Type	Target Antigen	Nucleic Acid	Route and Dose	Adjuvant	Results or Recruitment Status	Reference
Breast	Mammaglobin-A (Mam-A)	DNA	IM followed by EP, administered weeks 1, 4, and 8 by jet delivery device; unknown dose	None	Increased IFNγ-producing CD4+ T cells by vaccination	NCT00807781, [84]
				None	Significant increase in the number of antigen-specific CD8+ T cells	NCT00807781, [85]
	IGFBP-2, HER2, and IGF-1R	DNA	ID, administered every 28 days; 150 µg, 300 µg, or 600 µg	GM-CSF	Elevated Th1 responses by vaccination with grade 1/2 adverse events; 300 µg dose elicited persistent immune responses 6mo after vaccine administration	NCT02780401, [86]
	HER2 intracellular domain (ICD)	DNA	ID, administered once a month for three months; 10 µg, 100 µg, or 500 µg	GM-CSF	Elicited robust HER2-specific type 1 T-cell responses; Stronger immune responses in 100 µg and 500 µg doses in comparison to 10 µg dose	NCT00436254, [87]
	Several neoantigens	DNA	IM with electroporation; 4 mg administered 3 times, every 28 days	None	Increased IFNγ secreting CD8+ T cells	NCT02348320, [88]
	CD105, Yb-1, SOX2, CDH3, and MDM2	DNA	IV, administered once a month for 3 months, followed by boosters at 6 and 12 months	GM-CSF	Recruiting	NCT05455658
			ID, administered once a month for 3 months, with potential booster doses	GM-CSF	Active, not recruiting	NCT02157051
Melanoma	Emm55	DNA	Intralesion; administered one time in up to 3 lesions; 0.1 mg/lesion	None	Several patients exhibited stable disease	NCT03655756, [89]
	gp75 (TYRP1)	DNA	IM; administered every three weeks for 5 vaccinations; 0.1 mg to 8 mg dose-escalation study	None	Completed (results not provided)	NCT00034554
	Tyrosinase	DNA	IM; administered every three weeks; 100 µg, 500 µg, or 1500 µg doses	None	Demonstrated vaccine safety and feasibility; CD8+ T cell responses detected in 7 patients; no correlation of immune response with respect to dose or treatment arm	NCT00698100, [90]
		DNA	IM by electroporation; administered every three weeks up to five immunizations; 0.2 mg, 0.5 mg, or 1.5 mg per injection	None	Immune responses specific to tyrosinase were observed in 6/15 patients; immune responses were only detected in 1.5 mg cohort	NCT00471133, [91]
		DNA	IN continuously over 96 h every 14 days; 200 µg, 400 µg, or 800 µg	None	Immune responses detected in 11/26 patients	NCT00023647, [92]
	GM-CSF DNA plus Tyrosinase and gp100 peptides	DNA	SC; administered monthly for a total of three immunizations; 100 µg, 400 µg, or 800 µg of GM-CSF-encoding DNA followed by SC delivery of peptides to the same site on days 5 or 6 post DNA administration	GM-CSF	CD8+ T cell responses against melanoma peptides detected in 42% of patients, no correlations between dose and T cell responses were observed	NCT00580060, [93]
	Tyrosinase and Melan-A (MART-1)	DNA	IN; continuous infusion on days 1–4 with boosters every 14 d up to four courses; 500 µg, 1000 µg, or 1500 µg	None	Completed (results not provided)	NCT00033228
	NY-ESO-1, MAGE-A3, tyrosinase, and TPTE	mRNA	IV; administered in 6 injections within 43 days or 8 injections within 64 days; dose escalation study with doses varying from 14.4 µg to 400 µg total RNA	None	Induced strong CD4+ and CD8+ T cell immunity against the vaccine antigens; T cells responses were not dose-dependent	NCT02410733, [94]
	gp100	DNA	ID or IM; once every 4 weeks up to four doses	IL-2	Completed (results not provided)	NCT00019448
		DNA	IM; human gp100 in weeks 1, 4, and 7, followed by mouse gp100 in weeks 10, 13, and 16, or the reverse sequence; dose escalation study	None	Completed (results not provided)	NCT00104845
	Mouse tyrosinase-related protein 2 (TYRP2)	DNA	IM; every 3 weeks for 6 injections; doses of 500 µg, 2000 µg, 4000 µg, or 8000 µg	None	Completed (results not provided)	NCT00680589
	Melanoma-associated antigens	mRNA	ID	GM-CSF	Completed (results not provided)	NCT00204516
	Naked TAAs of melanoma	mRNA	IN; 8 immunizations over 43–51 days; total RNA doses of 100 µg, 200 µg, 600 µg, or 1200 µg containing RBL001 plus RBL002	None	Completed (results not provided)	NCT01684241
	Neoantigens (IVAC MUTANOME1)	mRNA	IN	None	Induced antigen-specific immune responses	NCT02035956, [95]
Prostate	PAP (pTVG-HP)	DNA	ID; 6 immunizations administered biweekly; 100 µg, 500 µg, or 1500 µg doses	GM-CSF	PAP-specific T-cell responses detected; responses were not correlated with treatment doses	NCT00582140, [96]
		DNA	ID; 100 µg; administered biweekly for 6 immunizations, then every 3 months until progression (Arm 1) or biweekly for 6 doses then administered every 2 weeks, 4 weeks, or 3 months as determined by immune responses	GM-CSF		NCT00849121, [97]
		DNA	ID; 100 µg; administered biweekly for 6 immunizations, then every 3 months for two years total treatment	GM-CSF		NCT01341652, [98]
	Androgen receptor ligand-binding domain (pTVG-AR)	DNA	ID; administered in biweekly immunizations for 6 doses, then every 3 months up to 12 months (schedule 1) or weeks 0, 2, 12, 14, 24, 26, 36, 38, 48, and 50 (schedule 2)	With or without GM-CSF	Th1-type immunity to the AR LBD detected; immunological responses in patients treated biweekly for 6 doses followed by boosters every 3 months (schedule 1) were superior to patients treated on the intermittent biweekly schedule (schedule 2)	NCT02411786, [99]
	TAAs including PSA, PSCA, PSMA, STEAP1	mRNA	ID; Phase I: 256 µg, 640 µg, 1280 µg; Phase II: 1280 µg; up to 5 immunizations	None	Induction of antigen-specific CD4+ and CD8+ T cells	EudraCT 2008-003967-37, [100]
	Prostate Specific Antigen (PSA)	DNA	ID with electroporation; administered every 4 weeks for 5 months; doses of 50 µg to 1600 µg	None	PSA specific T cell detected	NCT00859729, [101]
Cervical	HPV E6 and E7 (VGX-3100)	DNA	IM with electroporation; 6 mg; administered weeks 0,4, and 12	None	Better histological regression observed compared to placebo	NCT01304524, [102]
	HPV E6 and E7 (GX-188E)	DNA	Electroporation; 1 mg, 2 mg, or 4 mg; administered weeks 0, 4, and 12	None	HPV-specific CD8 T-cell response detected; responses independent of dose	NCT01634503, [103]
		DNA	Electroporation; 1 mg, 2 mg, or 4 mg doses	None	Unknown status	NCT02100085
		DNA	IM with electroporation; weeks 0, 4, and 12; doses of 1 mg or 4 mg	None	Completed, Results not posted	NCT02139267
		DNA	Electroporation; 1 mg and 4 mg; administered three times	None	Unknown status	NCT02411019
		DNA	IM with electroporation; 1 mg administered at weeks 0,4, and 12	None	Unknown status	NCT02596243
	HPV E6 and E7	DNA	IM with electroporation; administered at weeks 0,4,8,12	None	Antibody responses against HPV oncoproteins detected; antigen specific T cells detected	NCT02172911, [104]
		DNA	IM with electroporation; 1 mg, 4 mg or 8 mg doses	None	Recruiting	NCT06276101
	HPV E6/E7 fusion protein	DNA	IM; 3 immunizations of 3 mg each; administered on weeks 0, 3, and 6 (cohort 1) or weeks 0, 4, and 8 (cohort 2)	None	HPV-specific T-cell responses detected; Stronger T cell responses observed in cohort 1	NCT02529930, [105]
	HPV E6, E7, and L2 linked with calreticulin	DNA	IM with electroporation; 0.3 mg, 1 mg, or 3 mg doses	None	Not yet recruiting	NCT04131413
	HPV E7	DNA	IM; 3 doses at 1 month intervals; 0.5 mg, 1 mg, or 3 mg doses	None	HPV E7-specific T-cell responses detected; regression instances detected in 3 mg cohort	NCT00121173, [106]
	HPV specific antigens (RG002)	mRNA	IM	None	Recruiting	NCT06273553
Multiple advanced solid tumors	NY-ESO-1	DNA	ID by particle-mediated epidermal delivery; administered weeks 1, 5, and 9; 4 µg or 8 µg	None	NY-ESO-1-specific CD4+ and CD8+ T cells detected; no difference in immune responses between 4 µg and 8 µg cohorts	NCT00199849
	Preferentially expressed antigen in melanoma (PRAME) and prostate-specific membrane antigen (PSMA)	DNA	ID; administered on days 29 and 32 for up to 9 months; 30 µg or 300 µg	None	Increased PRAME-specific and PSMA-specific T cell responses; no difference in immune responses between low and high peptide dose cohorts	NCT00423254, [107]
	CD105, Yb-1, SOX2, CDH3, and MDM2	DNA	ID; administered on day 14 for 3, 21-day cycles	GM-CSF	Active, not recruiting	NCT05242965
	NY-ESO-1, MAGE-C1, MAGE-C2, Survivin, and TPBG	mRNA	ID; administered at weeks 1, 3, 7, 11, or at 1, 2, 3, 5, and 7; 400 µg, 800 µg, or 1600 µg total	None	Induction of immune response against five antigens; dosage did not cause significant differences in immune response	NCT00923312, [108]
Non-Small Cell Lung	Neoantigens	mRNA	SC	None	Recruiting	NCT03908671
	VEGFR-2	DNA	Oral; administered on days 1, 3, 5, and 7, then 4-weekly single doses every 4 weeks; 10^6^ or 10^7^ CFU	None	VEGFR-2 specific T cell response detected	NCT02718443, [109]
	IE-1, pp65, gB	DNA	IM with electroporation; 8 mg	None	Completed (Results not provided)	NCT05698199
	whole tumor mRNA, pp65, LAMP	mRNA	IV; administered every 2 weeks for 3 doses following radiation, then 12 doses monthly	None	Recruiting	NCT04573140
	pp65	mRNA	IV; 3 doses administered before or after tumor biopsy/resection	None	Recruiting	NCT06389591
	Neoantigens	DNA	IM; administered on days 1, 22, and 43 of cycle 1 and then day 1 of each subsequent cycle	Plasmid encoded IL-12	Active, not recruiting	NCT04015700
Glioblastoma	Tumor-associated antigens	mRNA	IM; administered on days 1, 8, 15, 29, 43, 57, and 71; 6 µg, 12 µg, 25 µg, 50 µg, 100 µg	None	Active, not recruiting	NCT05938387

**Table 3:** mRNA and DNA vaccines administered as parts of combination therapies in human clinical trials.

Cancer Type	Target Antigen	Nucleic Acid	Route and Dose	Combination Therapy	Results or Recruitment Status	Reference
Breast	Mammaglobin-A (Mam-A)	DNA	IM; administered every 28 days for 3 months; 4 mg	Neoadjuvant endocrine therapy	Active, not recruiting	NCT02204098
	IGFBP-2, HER2, and IGF-1R	DNA	ID; administered on day 13 and repeated up to 3 times, every 21 days, in the absence of disease progression	Paclitaxel (Chemo), trastuzumab and pertuzumab (anti-HER2)	Recruiting	NCT04329065
	HER2 intracellular domain (ICD)	mRNA	IV; administered every 2 weeks for 3 injections total; 4 × 10^8^ IU	Pembrolizumab (anti-PD-1)	Recruiting	NCT03632941
	Neoantigens	DNA	IM; administered every 28 days ± 7 days, with at least 21 days between injections by electroporation device; 4 mg	Durvalumab (anti-PD-L1)	Active, not recruiting	NCT03199040
				Chemotherapy and Pembrolizumab (anti-PD-1)	Recruiting	NCT06631092
	Shared TAAs and neoantigens	mRNA	IV	Surgery and adjuvant chemotherapy	Recruiting	NCT02316457
Melanoma	gp100 and TRP-2	DNA	IM; administered up to 11 times over 85 weeks using needle-free injection device	Nivolumab (Opdivo) and Ipilimumab (Yervoy)	Recruiting	NCT04079166
	Neoantigens	mRNA	IM; administered every 21 days up to 9 doses	Pembrolizumab	Prolonged recurrence-free survival in patients receiving vaccine plus Pembrolizumab versus Pembrolizumab alone; greater distant metastasis-free survival in combination therapy treated patients	NCT03897881 [140]
Colorectal	Oncoprotein MYB	DNA	ID; administered weekly for 6 weeks; 0.1 mg, 0.5 mg, or 1.0 mg	Tetanus toxoid peptides and anti-PD-1	Completed (result not provided)	NCT03287427 [141]
Prostate	PAP (pTVG-HP)	DNA	ID; administered bi-weekly for 4 weeks; 100 µg	Sipuleucel-T	Higher titer antibody responses to PAP detected	NCT01706458 [142]
		DNA	ID; administered every 2 weeks or every 3 weeks; 100 µg	Pembrolizumab (anti-PD-1)	PAP-specific T cells detected	NCT02499835 [143]
		DNA	ID; administered every 2 weeks for 6 vaccinations and then every 4 weeks for 9 vaccinations; 100 µg	Nivolumab (anti-PD-1)	PAP specific T cell responses detected with prolonged time to disease progression	NCT03600350 [144]
	PAP (pTVG-HP), Androgen receptor ligand-binding domain (pTVG-AR)	DNA	ID; administered on days 1 and 8 of 21 day cycles; 100 µg	Pembrolizumab (anti-PD-1)	Completed (results not provided)	NCT04090528
	Androgen receptor ligand-binding domain (pTVG-AR)	DNA	ID; administered weekly for 7 weeks; 100 µg	Degarelix (Androgen deprivation therapy), Cemiplimab (anti-PD-1), Fianlimab (anti-LAG-3)	Recruiting	NCT04989946
	PSA	DNA	SC; administered monthly	Flutamide (AR blockade)	flutamide + vaccine did not further improve patient outcome	NCT00450463 [145]
	Three prostate cancer-specific antigens (SL-T10)	DNA	IM; multiple injections administered; 3 mg or 6 mg	GX-I7 (T-cell growth factor), Pembrolizumab (anti-PD-1)	Recruiting	NCT06344715
	Neoantigens	DNA	IM, followed by EP; administered starting week 21 for a total of 6 treatments every 28 days; 4 mg	Nivolumab (anti-PD-1)/ipilimumab (anti-CTLA-4) and PROSTVAC	Completed (results not provided)	NCT03532217
	PSMA	DNA	IM (without EP): 800 µg, 1600 µg, or 3200 µg; IM (with EP): 400 µg, 800 µg, or 1600 µg; administered at weeks 0, 4, 8, 24, and 48	Fragment C of tetanus toxin	Antigen-specific CD4+ and CD8+ T cells detected; Stronger immune response with IM administration with EP	[124]
Glioblastoma	Wilms tumor gene-1 (WT1), PSMA, hTERT	DNA	IM followed by EP; administered starting on day 0 and every 3 weeks for 4 doses, then every 9 weeks until disease progression; 3 mg	INO-9012 (human IL12), cemiplimab, temozolomide, radiation	Increased antigen specific T cell populations	NCT03491683 [146]
	VEGFR-2	DNA	Oral; administered on days 1, 3, 5, 7 every 4 weeks; 10^6^ or 10^7^ CFU	anti-PD-L1	Increased number of intratumoral CD8+ T-cells detected	NCT03750071 [147]
	Neoantigens	DNA	IM with electroporation; administered once every 28 days for up to 6 doses	Retifanlimab (anti-PD-1)	Recruiting	NCT05743595
Pancreatic	mutant KRAS	mRNA	IM	Toripalimab (anti-PD-1)	Recruiting	NCT06577532
	Neoantigens	mRNA	Unknown route; administered 9 weeks post-tumor resection	Camrelizumab (anti-PD-1), Gemcitabine, Abraxane	Recruiting	NCT06326736
		mRNA	Unknown route; administered 12 weeks post-tumor resection	Ipilimumab (anti-CTLA-4), gemcitabine, capecitabine	Not yet recruiting	NCT06353646
		mRNA	Unknown route	Anti-PD-1	Recruiting	NCT06496373
		mRNA	Unknown route	Adebrelimab (anti-PD-L1)	Not yet recruiting	NCT06156267
		mRNA	IV; 25 µg administered weekly beginning 9 weeks post tumor resection plus booster doses at weeks 17 and 46	Atezolizumab (Anti-PD-L1), mFOLFIRINOX (chemotherapy)	50% of patients had detectable T cell responses to at least one neoantigen; vaccine-induced T cell clonal expansion detected in all immunological responders; longer recurrence free survival in immunological responders; persistent, high-avidity T cell clones detected	NCT04161755 [148,149]
Cervical	HPV E6 and E7 (GX-188E)	DNA	IM with electroporation; administered 3 times; 1 mg	GX-17 (IL-7 fused to hyFc), Imiquimod	Unknown status	NCT03206138
	HPV E6 and E7 (GX-188E)	DNA	IM; administered on days 1 and 29; 3 mg	Imiquimod, TA-HPV(recombinant vaccinia virus expressing HPV16/18 E6-E7 fusion proteins)	Half of the patients cleared their lesions	NCT00788164 [150]
		DNA	ID: 8 µg or 16 µg IM: 1 mg or 3 mg Intralesional: 1 mg or 3 mg Administered at weeks 0, 4, and 8	Imiquimod	Increased CD8+ T cell infiltration in cervical dysplastic epithelium in intralesional cohort	NCT00988559 [151]
Ovarian	p62/SQSTM1	DNA	IM; 2.5 mg; administered weekly	Gemcitabine	Higher objective response rate in patients receiving combination treatment of DNA vaccine and chemo versus chemo alone; greater number of partial responders and patients with stable disease in combination group; longer progression-free survival in combination treatment group	NCT05979298 [152]
Non-Small Cell Lung	Neoantigens	DNA	IM with electroporation; administered once every 4 weeks for 6 cycles	Durvalumab (anti-PD-L1)	Recruiting	NCT04397003
	NY-ESO-1, MAGE-C1, MAGE-C2, survivin, 5T4, and MUC-1	mRNA	ID; administered on days 1, 8, 15, 36, and 57, or days 1, 8, 15, 29, 43, and 57; 320 µg	Local radiation	Induction of immune response against six antigens	[153]
	NY-ESO-1, MAGE-C1, MAGE-C2, survivin, 5T4, and MUC-1	mRNA	ID; each component administered twice for a total of 12 vaccinations; 80 µg	Durvalumab (anti-PD-L1), Tremelimumab (anti-CTLA-4)	Completed (result not provided)	NCT03164772
	Neoantigens	mRNA	Unknown route	Adebrelimab (anti-PD-L1)	Not yet recruiting	NCT06685653
	Neoantigens	mRNA	Unknown route	Adebrelimab (anti-PD-L1)	Not yet recruiting	NCT06735508
	KRAS mutants (G12D, G12V, G12C, G13D)	mRNA	IM; administered once every 3 weeks for 9 doses; 1 mg	Pembrolizumab (anti-PD-1)	Completed (results not provided)	NCT03948763
	Tumor mRNA	mRNA	IV; administered 7–14 days after last dose of priming vaccine, then every two weeks for 2 doses, and then monthly for 9 doses	pp65 RNA-LP	Not yet recruiting	NCT05660408
